# Serum Zinc Concentrations and Dietary Zinc Adequacy in Children and Adolescents with Chronic Conditions: An Exploratory Secondary Analysis

**DOI:** 10.3390/nu18193245

**Published:** 2026-10-01

**Authors:** Marlene Fabiola Escobedo-Monge, José Manuel Marugán-Miguelsanz, Joaquín Parodi Román, María Antonieta Escobedo-Monge, M. Pilar Bahillo-Curieses

**Affiliations:** 1 Department of Pediatrics, Faculty of Medicine, University of Valladolid, Avenida Ramón y Cajal, 7, 47003 Valladolid, Spain; jmarugan@saludcastillayleon.es (J.M.M.-M.); mpbahillo@saludcastillayleon.es (M.P.B.-C.); 2 International Cooperation for Health and Social Development (CIDESS) Group, University of Las Palmas de Gran Canaria, Calle Juan de Quesada, 30, 35001 Las Palmas de Gran Canaria, Spain; 3 Valladolid Health Research Institute (IBioVALL), C. Rondilla Sta. Teresa, 47010 Valladolid, Spain; 4 Section of Gastroenterology and Pediatric Nutrition, University Clinical Hospital of Valladolid, Avenida Ramón y Cajal, 3, 47003 Valladolid, Spain; 5 Science Faculty, University of Cadiz, Paseo de Carlos III, 28, 11003 Cadiz, Spain; joaquin_parodi@yahoo.es; 6 Department of Chemistry, Science Faculty, University of Burgos, Plaza Misael Bañuelos SN, 09001 Burgos, Spain; antoitalia777@gmail.com

**Keywords:** deficient zinc intake, low/intermediate zinc deficiency

## Abstract

**Background/Objectives:** This secondary analysis evaluated the discordance between dietary adequacy and serum zinc levels in children and adolescents with chronic diseases, exploring the nutritional and biochemical traits of patients within an intermediate serum zinc range. **Methods:** Serum zinc was measured via atomic absorption spectrophotometry, and dietary tracking was conducted using a prospective 72 h food record. Subgroups were classified using Japanese Society of Clinical Nutrition thresholds (<60, 60–<80, and ≥80 μg/dL), treating the 60–<80 μg/dL band strictly as an operational, exploratory classification rather than a validated pediatric diagnostic standard. **Results:** Among the 90 participants, 31% fell into the 60–<80 μg/dL intermediate serum range and 61% exhibited inadequate dietary intake. Status discordance was frequent: 41% presented with inadequate dietary intake despite maintaining normal serum levels (≥80 μg/dL), whereas 11% had intermediate serum levels despite achieving adequate dietary intake. There was no significant association between serum zinc concentration and dietary zinc intake. Operationally classified intermediate-range participants demonstrated several nutritional and biochemical differences, notably showing higher intakes of vitamins B6, D, and E, elevated triglycerides, ESR, and IgG3, alongside lower hemoglobin. **Conclusions:** Dietary adequacy and serum zinc levels are frequently discordant, reinforcing that these metrics serve as complementary rather than interchangeable indicators of zinc status. The distinct profiles observed within the operational 60–<80 μg/dL category provide candidate relationships that warrant future targeted investigation.

## 1. Introduction

Zinc is an essential trace element required for normal growth, development, and physiological homeostasis. It has structural, catalytic, and regulatory roles through its involvement in proteins and enzymes governing cell proliferation, differentiation, apoptosis, migration, neuronal development, immune responses, and intracellular signaling [1,2]. Disruption of zinc homeostasis may impair redox balance and increase oxidative stress, affecting multiple systems, including the immune, nervous, and endocrine systems [3,4]. Altered zinc status has also been associated with infectious, metabolic, neurodegenerative, and malignant diseases, while zinc deficiency may contribute to disease development through epigenetic mechanisms involving DNA methylation, histone modifications, and microRNA regulation [5,6,7].

Zinc deficiency has a multifactorial etiology, primarily related to inadequate dietary intake but also to impaired absorption, increased endogenous losses, and increased physiological requirements during growth and illness [8,9]. It ranges from severe deficiency, typically associated with inherited disorders, malabsorption, chronic disease, or prolonged zinc-deficient parenteral nutrition, to marginal or subclinical insufficiency, which may occur without overt clinical manifestations [8]. Marginal zinc insufficiency is particularly difficult to identify because changes in zinc status may occur without characteristic clinical signs or markedly reduced serum zinc concentrations [9]. Although severe deficiency is uncommon in high-income countries, mild-to-moderate insufficiency may remain relevant in individuals with increased requirements, restrictive diets, or chronic diseases [10,11]. These considerations highlight the limitations of relying on a single indicator of zinc nutritional status.

Our previous studies showed that inadequate dietary zinc intake may occur without hypozincemia, with approximately 41% of patients with cystic fibrosis estimated to be at increased risk of inadequate zinc status [12]. We subsequently demonstrated that serum zinc concentrations could remain within the reference range despite dietary zinc intake below the selected reference level and that serum zinc was not significantly associated with dietary zinc intake [13]. These findings suggest that dietary zinc intake and serum zinc concentration may provide complementary rather than interchangeable information on zinc status. Therefore, in this secondary analysis, our primary objective was to characterize the discordance between dietary zinc adequacy and serum zinc concentration in children and adolescents with chronic diseases. Our secondary objective was to explore the clinical, anthropometric, nutritional, and biochemical characteristics associated with serum zinc concentration and dietary zinc intake within predefined serum-zinc categories.

## 2. Materials and Methods

### 2.1. Study Site, Design, and Participants

Designed as a secondary data analysis rather than an independent cohort, this study utilizes clinical, nutritional, and biochemical data collected between March 2002 and September 2003 at the Nutrition Unit of the Department of Pediatrics, University Clinical Hospital of Valladolid. This historical dataset was previously described in distinct studies evaluating zinc nutritional status in children with cystic fibrosis in 2019 [12] and children with chronic diseases in 2021 [13].

**Figure 1:**
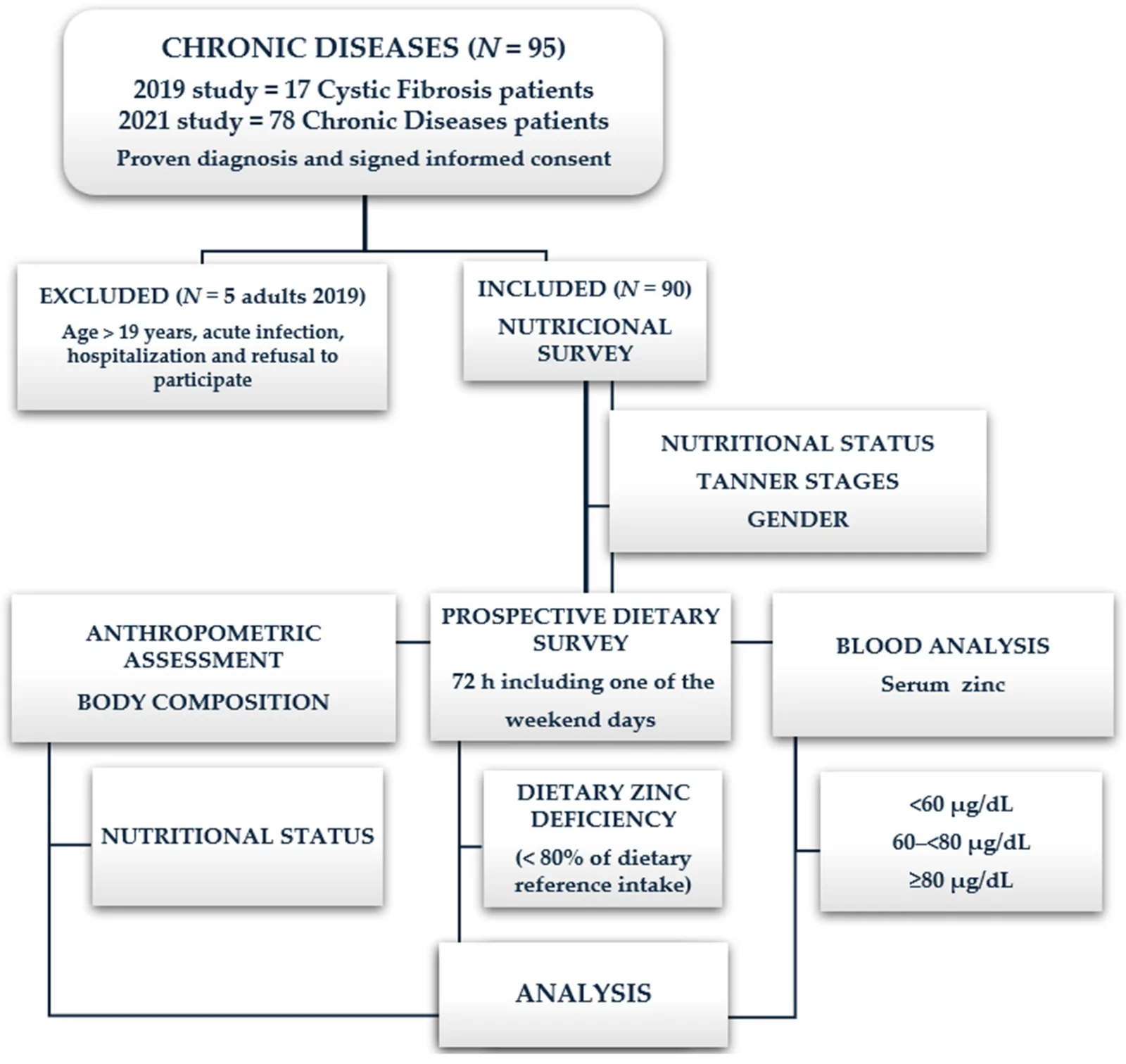
Flow diagram of participant selection for the present secondary analysis (*n* = 90).

The present analysis addresses a novel set of research questions by jointly classifying dietary zinc adequacy and serum zinc concentration, evaluating the distribution of an operational intermediate serum-zinc tier (60–<80 µg/dL), and exploring the associated clinical, anthropometric, and biochemical characteristics within predefined serum-zinc strata. The study population comprised children and adolescents aged ≥1 and <20 years with chronic conditions who had available dietary and serum zinc metrics. While specific baseline data, including the underlying chronic disease type and disease duration (months from diagnosis to enrollment), were recorded for all participants, patients presenting with acute infections, current hospitalization, or a refusal to participate were systematically excluded. The selection of participants included in the present secondary analysis is shown in Figure 1.

### 2.2. Ethical Considerations

The original study protocol was approved by the Ethics Committee of the University Clinical Hospital of Valladolid (INSALUD-Valladolid, 14 February 2002) and conducted in accordance with the Declaration of Helsinki. Written informed consent was obtained from the parents or legal guardians of all participants before enrollment.

### 2.3. Assessment of Phenotypical Characteristics

Demographic information, including age and sex, was obtained during the nutritional assessment. Trained personnel performed standardized anthropometric measurements, including weight, height, waist, hip, wrist, and mid-upper arm circumferences. Weight-for-age, height-for-age, weight-for-height, BMI, and mid-arm muscle area (MAMA) indices and Z-scores were calculated using Frisancho reference standards [14] and Fundación Orbegozo growth charts [15]. Skinfold thickness was measured at the triceps, biceps, subscapular, and suprailiac sites using a Holtain Skinfold Caliper. Body composition, including fat-free mass (FFM) and fat mass (FM), was assessed by anthropometry and bioelectrical impedance analysis (BIA) using the RJL BIA-101 analyzer (RJL Systems, Detroit, MI, USA).

### 2.4. Serum Zinc Measurement and Classification

Serum zinc concentrations were measured in fasting blood samples at the Department of Clinical Chemistry of the University of Valladolid using atomic absorption spectrophotometry (Philips PU9400), following established analytical procedures [16]. Measurements were performed in triplicate to ensure analytical accuracy and reproducibility. Participants were classified according to the serum zinc thresholds proposed in the 2018 Practical Guideline for Zinc Deficiency of the Japanese Society of Clinical Nutrition: zinc deficiency (<60 μg/dL), intermediate zinc concentrations (≥60 to <80 μg/dL), and normal zinc status (≥80 μg/dL) [17,18,19]. Because these thresholds were developed for adults and serum zinc may vary with age, sex, sampling time, nutritional status, and inflammation, the 60–<80 μg/dL category was used as an operational exploratory classification rather than a validated diagnostic definition of marginal zinc deficiency in children and adolescents. All participants also underwent complete blood count and routine biochemical analyses using standardized laboratory methods.

### 2.5. Dietary Assessment and Calculation of Zinc Intake/Adequacy

Dietary intake was assessed using a prospective 72 h food record comprising two weekdays and one weekend day, completed during the week before blood sampling. Daily energy, macronutrient, fiber, vitamin, and mineral intakes, including zinc, were calculated using the Mataix Food and Health software [20] and expressed as percentages of the applicable Spanish dietary reference values [21]. For this secondary analysis, dietary zinc intake below 80% of the selected reference value was used as an operational criterion for lower dietary zinc intake. This threshold was selected for consistency with the original dataset and was used solely for classification; it should not be interpreted as a validated diagnostic threshold for zinc deficiency or as equivalent to an EAR-based assessment of individual inadequacy. No participants received routine vitamin or mineral supplementation, except patients with cystic fibrosis, who received vitamins A, D, E, and K according to standard clinical practice.

### 2.6. Clinical Evaluation

During the clinical assessment, the researchers evaluated participants for signs and symptoms that could indicate impaired zinc status. They focused specifically on manifestations classically tied to zinc deficiency, such as dermatitis, cognitive dysfunction, night blindness, anorexia, delayed wound healing, hypogonadism, and compromised immune function [3,22].

### 2.7. Statistical Analysis

The study evaluated serum zinc concentration and dietary zinc intake/adequacy as primary outcomes alongside secondary anthropometric, biochemical, dietary, and body-composition variables, with participants stratified into children (≤10 years) and adolescents (>10 years). Following data distribution assessment via the Kolmogorov–Smirnov test, appropriate parametric or non-parametric tests (including *t*-test, ANOVA, Pearson/Spearman correlation, Mann–Whitney U, and Kruskal–Wallis) were selected to analyze baseline variables. The primary analysis examined classification concordance using an exact Fisher–Freeman–Halton test for a 3 × 2 contingency table, crossing three ordinal serum-zinc tiers (<60, 60–<80, and ≥80 µg/dL) with two dietary-zinc categories.

Correlation analyses were conducted separately within predefined serum-zinc strata, utilizing the Benjamini–Hochberg procedure to control the false discovery rate (FDR) across four prespecified testing families. Each testing family comprised 134 correlations (evaluating 135 total variables excluding the focal outcome), where unadjusted *p*-values were converted into FDR-adjusted q-values with a two-sided (q < 0.05) denoting statistical significance. Biologically relevant or significantly correlated variables were subsequently processed using exploratory single-predictor linear regression models in IBM SPSS Statistics version 29.0; however, due to the small intermediate subgroup size (*n* = 28) and a lack of simultaneous multivariable adjustments for clinical confounders like inflammation, treatment, or underlying disease, these regression coefficients (significant at a two-sided (*p* < 0.05) are strictly hypothesis-generating rather than definitive predictors.

## 3. Results

This study is a secondary analysis of a historical pediatric nutritional database that previously generated distinct publications in 2019 and 2021 [12,13], evaluating a final pediatric cohort of 90 participants aged 1 to 19 years (mean: 9.6 ± 4.8 years; median: 10.3 years) with a distribution of 49 females (54.4%) and 41 males (45.6%). The population consists of 78 participants (86.7%) from the 2021 chronic-disease cohort [13] and 12 pediatric participants (13.3%) with cystic fibrosis from the 2019 cohort [12], having excluded five individuals aged ≥ 20 years from the original cystic fibrosis dataset to maintain a strict pediatric focus. Stratified by age, the cohort includes 49 children (≤10 years) and 41 adolescents (>10 years), among whom short stature was identified in 16 of 90 participants (17.8%), including 7 of 18 children under 5 years old (38.9%) who met the clinical criteria for stunted growth.

Table 1 shows the distribution of dietary zinc and serum zinc concentration by categories. This cohort demonstrated a 53.3% overall discordance rate between dietary and biochemical classifications, with 55 participants (61.1%) showing low dietary intake (<80%) and 28 (31.1%) falling within the intermediate serum range (60–<80 µg/dL). Retaining the complete three-level serum classification against the dietary tiers revealed no statistically significant association via the Fisher–Freeman–Halton exact test (*p* = 0.466), confirming that these cross-classifications are descriptive artifacts of operational thresholds rather than a true diagnostic relationship. The single patient with serum zinc <60 μg/dL was excluded from subsequent subgroup analyses, reducing the sub-analysis population to 89 participants.

Descriptive tracking revealed a median serum zinc concentration of 86.8 μg/dL and a median dietary intake of 67.3% of the DRI, with neither metric differing significantly by sex or age. Within the intermediate subgroup, potential clinical signs appeared with variable frequencies (such as rough skin and dermatitis with alopecia), while feeding behavioral disturbances like lack of satiety (*n* = 27) and food refusal (*n* = 16) were highly prevalent. Group comparisons presented in Table 2 were exploratory and are reported using nominal, unadjusted *p*-values for multiple comparisons. Because the baseline group comparisons used nominal, unadjusted *p*-values across multiple variables, the resulting nominal differences in vitamins B6, D, and E intake, triglycerides, ESR, and IgG3 must be interpreted cautiously as purely exploratory and hypothesis-generating.

Table 3 shows the distribution of serum zinc categories and dietary zinc adequacy. Serum zinc concentrations ≥ 80 μg/dL were more common than concentrations of 60–<80 μg/dL across most disease groups, whereas inadequate dietary zinc intake was observed across several diagnostic categories. The proportion of participants with serum zinc concentrations of 60–<80 μg/dL was highest in kidney disease (5/7, 71.4%) and undernutrition (7/13, 53.8%). Inadequate dietary zinc intake was particularly common among participants with encephalopathies (8/8), eating disorders (4/4), and syndromic diseases (8/9). Overall, 61/89 participants (68.5%) had serum zinc concentrations ≥80 μg/dL, whereas 28/89 (31.5%) had concentrations of 60–<80 μg/dL. Inadequate dietary zinc intake was identified in 55/89 participants (61.8%), compared with 34/89 (38.2%) who met the predefined adequacy threshold.

Table 4 summarizes the correlations between serum zinc concentration or dietary zinc intake and the nutritional and biochemical variables evaluated within the serum-zinc subgroups. The Benjamini–Hochberg procedure was applied separately within four prespecified testing families defined by serum-zinc subgroup and outcome variable, with 134 correlations evaluated in each family. After FDR adjustment, three associations remained statistically significant. In participants in the 60–<80 μg/dL subgroup, dietary zinc intake was positively correlated with vitamin D intake (r = 0.640, q = 0.043). In participants with serum zinc ≥ 80 μg/dL, dietary zinc intake was positively correlated with niacin intake (r = 0.489, q = 0.004) and magnesium intake (r = 0.517, q = 0.003). No correlation involving serum zinc concentration remained statistically significant after FDR correction. The complete set of tested correlations, including unadjusted *p*-values and FDR-adjusted q-values, is provided in Supplementary Table S1.

In relation to Table 5, exploratory single-predictor linear regression analyses were performed to characterize potential associations with serum zinc concentration or dietary zinc intake. Due to the limited sample size—particularly within the 60–<80 μg/dL intermediate subgroup (*n* = 28)—predictors were selected a priori based on biological relevance and univariable findings to preserve model parsimony. The resulting models identified several candidate associations, but because they utilized unadjusted *p*-values for multiple comparisons and included only a single predictor without controlling for clinical heterogeneity, they cannot establish independent determinants. Consequently, these findings must be interpreted strictly as exploratory and hypothesis-generating associations requiring prospective confirmation in larger, more homogeneous pediatric cohorts.

## 4. Discussion

This study is a secondary, exploratory analysis of an overlapping pediatric cohort with chronic diseases that extends previous findings by evaluating the unique characteristics of an intermediate serum zinc range. A critical outcome is the substantial discordance between dietary zinc intake and serum zinc concentration, demonstrating that these two metrics offer complementary rather than interchangeable data regarding an individual’s zinc status. Additionally, exploratory regression analyses identified candidate nutritional and biochemical factors associated with zinc parameters, though these insights remain strictly hypothesis-generating rather than confirmatory.

Operationally, 28 participants (31.5%) presented with serum zinc concentrations within the predefined intermediate range of 60–<80 μg/dL. Although the Japanese Society of Clinical Nutrition classifies this specific range as “marginal zinc deficiency,” this guideline was developed primarily for adults and is not a validated diagnostic threshold for children [18]. Therefore, the authors emphasize that this subgroup must be interpreted as an operational intermediate range rather than a true 31.5% prevalence rate of pediatric marginal zinc deficiency.

### 4.1. Serum Zinc and Dietary Zinc Are Discordant

Evaluating four combinations of serum zinc concentration and dietary adequacy, this portion of the study confirms that a frequent occurrence of dietary inadequacy and a lack of significant association between intake and serum levels reinforce the limitations of serum zinc as a standalone biomarker [23]. This marked discordance demonstrates that dietary intake and serum concentrations provide complementary rather than interchangeable data, as individual serum levels are heavily influenced by non-dietary variables such as age, sex, sampling time, inflammation, infection, and metabolic alterations [23]. Even though a previous systematic review in children identified a non-proportional 9% increase in serum/plasma levels for every doubling of zinc intake, the lack of concordance here reflects that intake alone cannot capture individual concentrations due to homeostatic factors like absorption rates, endogenous losses, physiological needs, and dietary bioavailability [24,25].

### 4.2. The 60–<80 μg/dL Group Has Potentially Distinctive Characteristics

Pediatric participants within the intermediate serum zinc range (60–<80 μg/dL) differed from those with normal levels (≥80 μg/dL) by exhibiting higher intakes of vitamins B6, D, and E, elevated triglycerides, erythrocyte sedimentation rates, and IgG3 levels, alongside lower hemoglobin concentrations. These variances in inflammatory and hematological markers reinforce that serum zinc reflects complex physiological processes far beyond dietary exposure, meaning this diagnostically uncertain intermediate range must not be interpreted as a true prevalence rate of pediatric marginal zinc deficiency. Subgroup-specific complexities were further highlighted by an unexpected inverse association between wrist circumference—a marker of skeletal development and cardiometabolic risk [13,26]—and zinc levels within the intermediate group, contrasted with a significant association between dietary intake and estimated fat mass in the normal group (≥80 μg/dL). Rather than implying causation, these shifting relationships across different serum tiers may be driven by age-related variations in zinc requirements, body composition, underlying chronic disease, or residual confounding [27].

#### 4.2.1. Growth, Anthropometry, and Body Composition

Within the small subgroup of seven patients with stunted growth, three (42.9%) had intermediate serum zinc levels (60–<80 μg/dL), suggesting that children with impaired linear growth warrant attention regarding zinc status, though the sample size precludes any definitive inference regarding prevalence or risk [28]. Furthermore, an unexpected inverse relationship between serum zinc and height-for-age in this subgroup cannot establish causality or timing due to the study’s cross-sectional nature, and it likely reflects differences in age, body size, underlying chronic disease, or unmeasured confounding despite zinc’s established necessity for protein synthesis and pediatric growth velocity [29,30,31]. Additionally, a significant association between dietary zinc intake and anthropometrically estimated fat mass observed specifically within the normal zinc subgroup (≥80 μg/dL) does not prove that zinc intake directly determines fat mass. Prior literature has frequently connected zinc status to adiposity and distinct metabolic traits, though these outcomes vary heavily across different populations [32,33,34]. Because zinc intake is inherently tied to overall caloric consumption and macronutrient composition, this link likely reflects broader nutritional patterns, overall dietary quality, or baseline participant characteristics rather than an isolated, nutrient-specific effect.

#### 4.2.2. Zinc, Dietary Fat, and Lipid-Related Variables

Serum zinc significantly correlated with dietary fat intake within the intermediate group (60–<80 μg/dL), whereas lipid-related variables—including the LDL/HDL ratio, cardiovascular risk index, and LDL-cholesterol—associated significantly with serum zinc in the normal subgroup (≥80 μg/dL). While these correlations provide a plausible biological rationale for investigating the crosstalk between zinc homeostasis and lipid metabolism—particularly how dietary choices affect zinc availability and how zinc regulates adipocyte function [35,36]—the authors stress that these subgroup-specific findings do not prove a causal or bidirectional relationship with cardiovascular risk. An association with an adverse lipid profile does not mean inadequate zinc status is the cause, nor does it rule out that lipid characteristics independently coexist with factors influencing serum zinc.

#### 4.2.3. Zinc and Other Micronutrient Relationships

Subgroup-specific analyses revealed that dietary zinc intake positively correlated with vitamin D in the intermediate group (60–<80 μg/dL) and with niacin in the normal group (≥80 μg/dL); however, despite the biological plausibility of these nutrient-receptor and absorption interactions, the study’s cross-sectional design cannot prove direct biological links as these elements frequently share common food sources [9,37,38]. Similarly, positive correlations between dietary zinc and total magnesium intake across both groups align with historical data showing that zinc intake closely parallels overall dietary adequacy and nutrient density rather than acting independently [39]. While zinc, magnesium, and vitamin D collectively govern pediatric growth and bone metabolism, clinical supplementation trials cannot be directly extrapolated to these observational findings [40,41,42,43]. Finally, although vitamin D intake remained positively associated with zinc intake in exploratory regression modeling, the authors emphasize a cautious, hypothesis-generating interpretation due to the limited statistical power and intentionally parsimonious models necessitated by the small intermediate subgroup size (*n* = 28). Dietary zinc intake positively correlated with iron intake within the intermediate (60–<80 μg/dL) group. The positive alignment between dietary zinc and iron is easily explained by their shared food sources and overall dietary composition [44]. While both deficiencies can coexist and lead to anemia or impaired growth, and though high supplemental doses can cause absorption interference at the intestinal level, these cross-sectional findings must be viewed as a reflection of the intricate interplay among micronutrient status, inflammation, and chronic illness [45,46,47,48].

### 4.3. The 60–<80 μg/dL Group Analysis

The 60–<80 µg/dL serum zinc range must be interpreted with strict caution. Furthermore, a non-dichotomized analysis across the three categories (<60, 60–<80, and ≥80 µg/dL) failed to reveal a statistically significant association with dietary zinc adequacy. Therefore, the noted 53.3% discordance rate merely represents the proportion of participants whose dietary and biochemical indicators diverged under this classification system, rather than an estimate of true deficiency prevalence.

Interpreting the clinical differences among these subgroups is significantly complicated by the marked clinical heterogeneity of the cohort. Because these distinct conditions independently affect dietary intake, systemic inflammation, lipid metabolism, hematological parameters, and body composition, the observed subgroup differences cannot be directly or specifically attributed to zinc status itself. The study’s single-predictor regression models were not structured to adjust for this heterogeneity or to control for residual confounding driven by disease states, treatments, inflammation, or dietary patterns. Therefore, the observed correlations should not be seen as evidence that intermediate zinc levels cause these nutritional, anthropometric, or biochemical shifts.

Methodologically, this study is a secondary analysis of previously reported data and does not represent an independent replication cohort. Out of the 90 total participants evaluated, 78 were derived from an earlier chronic-disease cohort [13] and 12 from a previous cystic-fibrosis cohort [12]. The novel contribution of this analysis is its joint evaluation of dietary adequacy and serum concentrations to characterize the 60–<80 µg/dL subgroup across this combined pediatric population. Because the intermediate subgroup was limited to just 28 participants and evaluated via univariable models, the results serve solely as hypothesis-generating candidate relationships. Ultimately, these findings do not establish diagnostic accuracy, predict clinical outcomes, or prove that children with low or intermediate levels would benefit from zinc supplementation.

#### Limitations and Suggestions

The clinical utility and generalizability of this study are constrained by several distinct limitations. First, the dataset is historical (2002–2003), meaning the findings may not accurately reflect modern pediatric populations given major subsequent shifts in dietary trends, reference guidelines, and chronic disease management. Second, the cohort exhibits severe clinical heterogeneity across diverse underlying diagnoses, treatments, and inflammatory states that could not be fully controlled, making it impossible to attribute the observed variations exclusively to zinc status. Furthermore, the 60–<80 μg/dL range lacks pediatric validation because it was adapted from adult clinical criteria, meaning it serves only as an operational, exploratory classification rather than a verified diagnostic threshold for marginal zinc deficiency. Finally, statistical power was highly restricted due to the small intermediate subgroup (*n* = 28), and because the analyses involved multiple statistical comparisons, the resulting regression data must be interpreted as hypothesis-generating rather than confirmatory.

To resolve these limitations, future research must incorporate larger, more homogeneous pediatric cohorts alongside healthy comparison groups. Incorporating longitudinal study designs with repeated serum measurements will clarify whether intermediate zinc levels persist over time and if they reliably predict long-term clinical or nutritional outcomes. Future investigators should prioritize establishing age- and sex-specific pediatric reference ranges, standardizing blood sampling conditions, and thoroughly evaluating systemic inflammation and long-term dietary habits. Ultimately, prospective intervention studies are required to definitively demonstrate whether children presenting with persistently low or intermediate serum zinc levels derive measurable clinical benefit from targeted nutritional therapy or zinc supplementation.

## 5. Conclusions

This secondary analysis of children and adolescents with chronic diseases demonstrates a frequent discordance between dietary zinc intake and serum zinc concentration, indicating that these metrics capture distinct, non-interchangeable aspects of individual zinc status. The evaluated 60–<80 μg/dL serum-zinc category showed isolated associations with specific dietary, anthropometric, inflammatory, lipid, and hepatic biomarkers.

Overall, the study highlights the inherent difficulty of characterizing pediatric zinc profiles using a single metric. Ultimately, larger, contemporary longitudinal studies with standardized sampling and validated, age- and sex-specific reference ranges are required to determine whether combining these assessment tools improves diagnosis, clinical outcomes, or the targeted identification of children who would benefit from zinc supplementation.

## Figures and Tables

**Table 1:** Distribution of dietary zinc adequacy according to serum zinc concentration category (*n* = 90).

Serum Zinc Category	Dietary Zinc < 80%	Dietary Zinc ≥ 80%	Total (%)
<60 μg/dL (Low)	0	1	1 (1.1)
60–<80 μg/dL (Intermediate)	18	10	28 (31.1)
≥80 μg/dL (Normal)	37	24	61 (67.7)
Total (%)	55 (61.1)	35 (38.9)	90 (100)
Concordant lower dietary/intermediate serum category: 18/90 (20.0%) Low dietary intake with serum zinc ≥ 80 μg/dL: 37/90 (41.1%) Intermediate serum zinc with dietary intake ≥ 80%: 10/90 (11.1%) Serum zinc < 60 μg/dL with dietary intake ≥ 80%: 1/90 (1.1%)			

**Table 2:** Clinical, anthropometric, nutritional, and biochemical characteristics according to serum zinc category (*n* = 89).

Characteristics	Serum Zinc 60–<80 μg/dL	Serum Zinc ≥80 μg/dL	*p*-Value
	Mean ± SD	Mean ± SD	
Female, n (%)	18 (64.3%)	30 (49.2%)	
Age (years), Median (IQR)	10.8 (6.4–15.2)	10.2 (6.5–13.9)	0.994
Time of chronic diseases (months)	59 ± 52	78 ± 49	0.096
Dietary zinc intake (%DRI)	69 ± 33	73 ± 36	0.571
Vitamin B6 intake (%DRI), Median (IQR)	107 (75–139)	107 (74–140)	0.041
Vitamin D intake (%DRI)	246 ± 122	66 ± 108	0.012
Hemoglobin (g/dL)	12.9 ± 1.6	13.6 ± 1.0	0.036
Lymphocytes	3123 ± 1279	3130 ± 1426	0.984
C-reactive protein	3.7 ± 1.7	3.9 ± 1.8	0.543
Erythrocyte Sedimentation Rate (mm/h)	18.4 ± 10.0	12.8 ± 5.7	0.002
Albumin	4.2 ± 0.3	4.3 ± 0.3	0.364
Serum vitamin E (mg/dL)	19.4 ± 5.9	13.9 ± 4.8	<0.001
Triglycerides (mg/dL), Median (IQR)	79 (46–112)	65 (46–84)	0.019
Phosphatase alkaline, Median (IQR)	312 (163–460)	345 (182–527)	0.335
IgG3	47.0 ± 28.1	33.8 ± 18.6	0.025

Abbreviation: DRI: Dietary Reference Intake. ANOVA test was used.

**Table 3:** Distribution of serum zinc status and dietary zinc intake among chronic disease patients (*n* = 89).

Chronic Condition	Total *n*	Dietary Zinc ≥ 80%, *n*	Dietary Zinc < 80%, *n*	Serum Zinc ≥ 80 μg/dL, *n*	Serum Zinc 60–<80 μg/dL, *n*
Obesity	22	9	13	16	6
Cystic Fibrosis	11	9	2	8	3
Undernutrition	13	5	8	6	7
Syndromic diseases	9	1	8	8	1
Various diseases	7	3	4	5	2
Encephalopathies	8	0	8	7	1
Kidney diseases	7	3	4	2	5
Hyperlipidemias	4	2	2	2	2
IDDM	4	2	2	3	1
Eating Disorders	4	0	4	4	0
Total	89	34	55	61	28

Abbreviation: IDDM: Insulin-dependent diabetes mellitus.

**Table 4:** Correlations between dietary zinc intake and nutritional variables that remained significant after Benjamini–Hochberg false-discovery-rate correction according to serum-zinc category.

Serum-Zinc Subgroup *	Outcome	Variable	r	Unadjusted *p*-Value	BH-Adjusted q-Value
60–<80 μg/dL	Dietary zinc intake	Vitamin D (%DRI)	0.640	<0.001	0.043
≥80 μg/dL	Dietary zinc intake	Niacin (%DRI)	0.489	<0.001	0.004
≥80 μg/dL	Dietary zinc intake	Magnesium (%DRI)	0.517	<0.001	0.003

* Only associations that remained statistically significant after Benjamini–Hochberg false-discovery-rate correction are shown. Correlation coefficients are Pearson’s r. Unadjusted *p*-values are shown together with FDR-adjusted q-values. Each prespecified testing family comprised 134 correlations. A q-value < 0.05 was considered statistically significant. The complete set of tested correlations is provided in Supplementary Table S1.

**Table 5:** Exploratory linear regression models of serum zinc concentration and dietary zinc intake according to serum zinc category (60–<80 μg/dL, *n* = 28; ≥80 μg/dL, *n* = 61).

	Predictor	B	β	95 CI for B	*p*-Value	Adjusted R^2^	VIF
Serum zinc 60–<80 μg/dL							
Serum zinc	WC	−0.687	−0.558	−1.109 to −0.266	0.003	0.283	1.000
Zinc intake	Vitamin D (%DRI)	0.041	0.640	0.021 to 0.061	<0.001	0.386	1.000
Serum zinc ≥ 80 μg/dL							
Serum zinc	Risk index	4.586	0.394	1.771 to 7.402	0.002	0.140	1.000
	LDL/HDL ratio	4.759	0.369	1.611 to 7.907	0.004	0.121	1.000
	LDL-Cholesterol	0.119	0.342	0.033 to 0.205	0.008	0.101	1.000
Zinc intake	Fat mass by anthropometry	1.042	0.339	0.288 to 1.795	0.008	0.100	1.000
	Magnesium (%DRI)	0.487	0.517	0.277 to 0.698	<0.001	0.255	1.000
	Iron (%DRI)	0.094	0.397	0.037 to 0.151	0.002	0.143	1.000

Abbreviations: β, standardized regression coefficient; B, unstandardized regression coefficient; CI, confidence interval; VIF, variance inflation factor; DRI, Dietary Reference Intake; WC, wrist circumference; LDL, low-density lipoprotein; HDL, high-density lipoprotein. β represents the standardized regression coefficient, and 95% CIs are reported for B. Adjusted R^2^ represents the proportion of variance explained by the model after adjustment for the number of predictors. All models were exploratory and hypothesis-generating. Each regression model included one predictor and was fitted separately.

## Data Availability

The raw data supporting the conclusions of this article will be made available by the authors on request.

## Supplementary Materials

The following supporting information can be downloaded at: https://www.mdpi.com/article/10.3390/nu18193245/s1, Table S1: Complete Correlation Analyses.
